# Intestinal Intussusception Complicating an Undiagnosed Burkitt Lymphoma in a Pediatric Arab Patient

**DOI:** 10.7759/cureus.55949

**Published:** 2024-03-11

**Authors:** Marwa Alghenaim, Mohamed Awadh, Abdulrahman Alshafai, Abdulla Darwish

**Affiliations:** 1 Department of Pathology and Laboratory Medicine, Bahrain Defense Force (BDF) Royal Medical Services, Riffa, BHR; 2 Department of Pediatric Surgery, Bahrain Defense Force (BDF) Royal Medical Services, Riffa, BHR

**Keywords:** tumor markers, management guideline, imaging, chemotherapy, pediatric surgery, immunohistochemistry, histopathology, medical oncology, intussusception, burkitt’s lymphoma

## Abstract

Burkitt’s lymphoma (BL) is considered an aggressive form of a non-Hodgkin B-cell lymphoma, representing less than 5% of all pediatric malignancies and 30% of pediatric lymphomas. However, intestinal BL may present as a lead point, causing intussusception. Surgery continues to be the gold standard for the treatment and identification of localized tumors to ensure complete removal with proper margin. In this report, we describe a hidden BL presenting as intestinal intussusception in an eight-year-old Arab boy. A computed tomography (CT) scan of the abdomen revealed an ileoileal intussusception with multiple enlarged lymph nodes. The report discusses the role of histopathology, supported by immunohistochemistry studies, in establishing the diagnosis. It also covers the significance of proper laparoscopic surgery and chemotherapy in the management of this child.

## Introduction

Burkitt's lymphoma (BL) was initially described in 1958 by Dennis Burkitt [1]. It is considered an aggressive form of non-Hodgkin B-cell lymphoma, representing less than 5% of all pediatric malignancies and 30% of pediatric lymphomas [2]. Although gastrointestinal involvement is a typical finding in cases of sporadic BL, primary intestinal lymphomas are still a very unusual occurrence. In the presence of gastrointestinal involvement, the lesion may act as a lead point, causing intussusception [3,4]. The clinical presentation consists of abdominal discomfort, vomiting, and lower gastrointestinal hemorrhage [5,6]. Sometimes, it is possible to palpate an abdominal mass. However, in cases of recurrent intussusceptions, symptoms may remain for prolonged durations while being milder and more intermittent. In this approach, the tumor may progress asymptomatically while expanding in size, revealing itself only at a late stage when it begins to produce serious complications, such as intestinal obstruction, gastrointestinal hemorrhage, or even intestinal necrosis [7]. In this report, we describe a case of BL presenting as intussusception with intestinal obstruction in an eight-year-old Arab boy.

## Case presentation

An eight-year-old previously healthy boy of Arab descent reported to the emergency room with complaints of abdominal pain associated with vomiting for several months. The pain was not responding to analgesic medication, and the patient was empirically treated for Helicobacter pylori without any benefit. No history of abdominal distention, weight loss, fever, jaundice, or night sweats were reported. Medical, psychological, and family history were unremarkable.

The patient’s height was 135 cm and his weight was 50 kg. While in the emergency room, he was found to be normotensive and afebrile. The abdomen was soft on physical examination, with a palpable mass in the right paraumbilical region and no distension. Hepatosplenomegaly and lymphadenopathy were not observed.

Laboratory examinations revealed a C-reactive protein (CRP) value of 8.16 mg/L, and lactate dehydrogenase (LDH) was within the normal range. Complete blood counts, renal function, and liver function tests were within normal limits.

**Figure 1:**
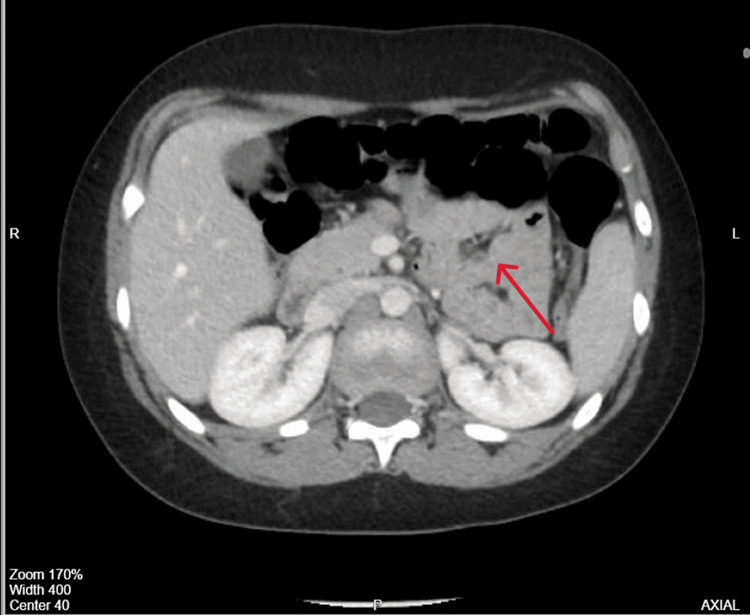
Computed tomography (CT) scan showing ileoileal bowel intussusception with multiple enlarged lymph nodes nearby. There is no free fluid or abdominal collection. The solid organs are unremarkable.

Ultrasonography (US) of the abdomen showed a persistent lengthy segment of bowel intussusception with a thickened bowel wall. A computed tomography (CT) scan of the abdomen revealed an ileoileal intussusception with multiple enlarged lymph nodes (Figure 1).

The child underwent an emergency laparoscopic reduction of the ileoileal intussusception. Following reduction, a suspicious mass was identified in the mid-ileum and the umbilical incision was extended to deliver the small bowel. A small bowel resection and anastomosis were performed with complete gross resection of the suspicious lesion.

**Figure 2:**
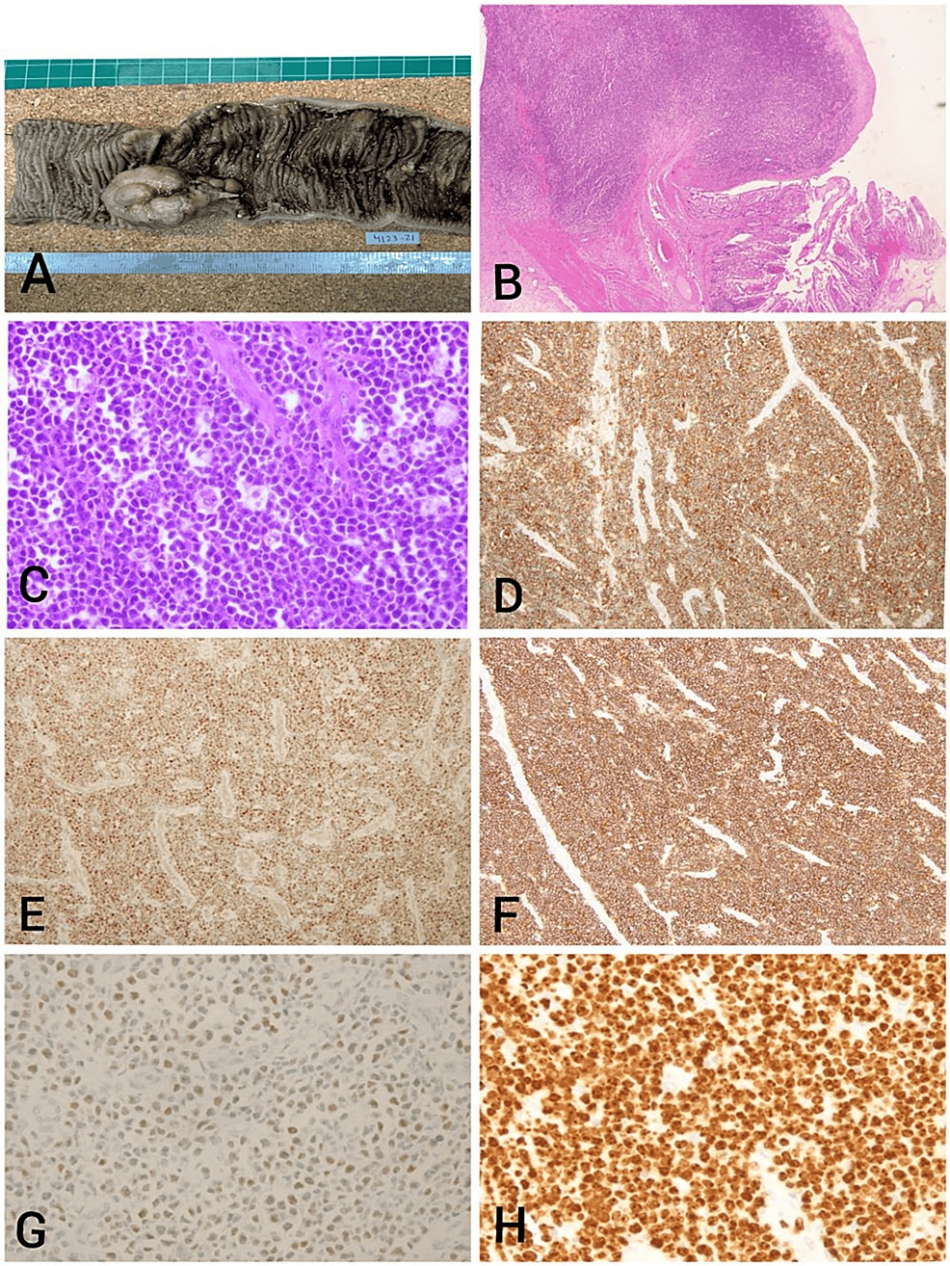
(A) Gross image of Burkitt’s lymphoma displaying a firm, grayish-white nodular tumor; (B) low-power microscopic view revealing a diffuse expansion of the mucosa and submucosa by the tumor (H&E stain); (C) high-power microscopic view depicting sheets of monotonous intermediate-size tumor cells exhibiting nuclear molding, numerous mitoses, and apoptosis (H&E stain); (D)-(G) the tumor cells are positive for CD20, BCL6, CD10, and MYC; (H) the tumor cells demonstrate a 100% reaction with the Ki-67 proliferative index.

A segment of the small bowel, measuring 20 cm x 3.5 cm, was partially opened at one end. Upon further opening, two firm greyish-brown polypoidal masses were observed. The larger one measured 3.5 x 3.5 cm, and the smaller one measured 1.5 x 1.5 cm. Additionally, a mass located 0.5 cm away from the main mass was revealed on the cut-open specimen (Figure 2A). Pathological examination showed a tumor composed of diffuse infiltration by sheets of monotonous intermediate-size malignant cells with a starry sky appearance (Figure 2B). The tumor showed frequent mitosis, apoptosis, and phagocytic tangible body macrophages (Figure 2C). An immunohistochemical study of the resected tumor showed positivity for CD20, CD10, and BCL6, whereas BCL2, CyclinD1, and CD21 were negative. CD5 and CD3 were positive in the reactive T-cells in the background (Figures 2D-2G). The proliferation index Ki-67 was approximately 100% (Figure 2H). The overall appearances confirm the diagnosis of BL.

Postoperatively, the patient had an uneventful recovery and was discharged home after surgery. The PET scan with chest CT was done, and both were negative. He was referred to the oncology department for evaluation and initiation of the chemotherapeutic treatment. The final diagnosis was BL (Group B intermediate risk), and the management was given according to the International guideline protocol. The patient has received five cycles of chemotherapy: one cycle of COP (Cyclophosphamide, Oncovin [Vincristine], Prednisone), followed by one cycle of COPDAM (Cyclophosphamide, Oncovin [Vincristine], Prednisolone, Adriamycin [Doxorubicin], Methotrexate), one cycle of RCOPDAM (Rituximab, Cyclophosphamide, Oncovin [Vincristine], Prednisolone, Adriamycin [Doxorubicin], Methotrexate), and two cycles of R-CYM (Rituximab, Cytarabine [Aracytine, Ara-C], Methotrexate). He responded very well to the treatment, and his disease remained in remission two years post-chemotherapy completion.

## Discussion

In children, primary tumors of the gastrointestinal (GI) tract constitute less than 5% of all pediatric tumors [8]. The disease's rarity and varying clinical presentation preclude early discovery when a cure is possible [9]. Hodgkin lymphoma (HL) and non-Hodgkin lymphoma (NHL) are the two main types of lymphomas, each having distinct clinical presentations and treatments. In children, high-grade NHL encompasses four categories: lymphoblastic lymphoma, BL, diffuse large B-cell (DLBCL), and anaplastic large-cell lymphoma [10]. BL is one of the most rapidly progressing cancers and the most prevalent subtype of NHL in children. The male-to-female ratio ranges from 1.3:1 to 8.8:1 for this condition. It occurs in individuals aged 0 to 20 years, with a median age of eight years, and approximately one-third of cases manifest between five and nine years [11].

Three subtypes of BL have been identified by the World Health Organization: endemic, sporadic, and immunodeficiency-associated [12]. The endemic (African) type primarily affects the maxilla and mandible, whereas the nonendemic (sporadic) type primarily affects the distal ileum, cecum, and mesentery [13]. Ninety-five percent of endemic instances are connected with the Epstein-Barr virus, compared to 15% of sporadic cases [13]. The immunodeficiency-associated type occurs most frequently in HIV patients, but can also manifest in allograft recipients and congenital immune deficiency diseases [12]. We believe that our patient is of sporadic type since he is of Arab descent with no immunodeficiency manifestations. Children typically exhibit nausea, vomiting, abdominal pain, and distension due to abdominal masses, intestinal obstruction from bowel compression or intussusceptions, or acute appendicitis [13]. Our patient presented with abdominal pain and vomiting as symptoms. The diagnosis of NHL is suspected based on US or CT scans but only confirmed by the histopathological examination [10]. In our case, both transabdominal US and abdominal CT suggested intussusception. Surgery continues to be essential in the treatment and identification of localized tumors to ensure complete removal with proper margin [14]. The advent of laparoscopy has allowed a less invasive approach to this illness with faster recovery and shorter hospital stays. The intussusception can often be gently reduced by laparoscopy followed by a limited extension of the umbilical wound and delivery of the resected mass.

BL is a mature B-cell lymphoma that expresses surface immunoglobulin (sIg) in addition to a spectrum of surface B-cell markers (CD19, CD20, and CD22), CD10, BCL6, CD38, CD77, and CD43 [15]. TCF3 or ID3 mutation is found in up to 70% of BL cases [15]. A reciprocal translocation between the *c-myc* gene and one of the immunoglobulin genes is also present in these lymphomas [16]. Treatment of BL is based on short-duration, dose-intensive systemic chemotherapy regimens, with various considerations to consider, including the tumor's high growth rate, the patient's age, and the risk of tumor lysis syndrome [2]. According to the International guideline for the management of mature B-cell NHL, the chemotherapy regimen for managing BL consists of the following chemotherapeutic agents: Cyclophosphamide, Oncovin (vincristine), prednisolone, Adriamycin (doxorubicin), methotrexate, with or without Rituximab. The specific combination used depends on the disease group (A, B, or C) and the risk categories (low, intermediate, and high) [17]. Our patient was given and followed chemotherapy protocol with excellent outcomes. However, the prognosis is based upon clinical and histopathologic staging, especially disease extent and chemotherapy response. In general, younger patients have a better prognosis. Moreover, cytogenetic abnormalities beyond the MYC translocation, such as deletion of 13q, gain of 7q, or complex cytogenetics, may be associated with a poorer prognosis. Poor response to cyclophosphamide, prednisolone, and vincristine, as well as evidence of spread to the central nervous system (CNS), and low CD4 count, is indicative of a poor outcome [16].

## Conclusions

Surgeons should be aware that BL can be considered in the differential diagnosis as a leading point if a child presents with intussusception. A multidisciplinary team with a proper management plan is necessary to address this type of malignancy, especially since BL shows an excellent chemotherapy response when following international guidelines.
